# Bioimpedimetric analysis in conjunction with growth dynamics to differentiate aggressiveness of cancer cells

**DOI:** 10.1038/s41598-017-18965-9

**Published:** 2018-01-15

**Authors:** Aditya Parekh, Debanjan Das, Subhayan Das, Santanu Dhara, Karabi Biswas, Mahitosh Mandal, Soumen Das

**Affiliations:** 1 School of Medical Science and Technology, IIT Kharagpur, West Bengal India; 2 Department of Electronics and Communications Engineering, DSPM IIIT, Naya Raipur, India; 3 Department of Electrical Engineering, IIT Kharagpur, West Bengal India

## Abstract

Determination of cancer aggressiveness is mainly assessed in tissues by looking at the grade of cancer. There is a lack of specific method to determine aggressiveness of cancer cells *in vitro*. In our present work, we have proposed a bio-impedance based non-invasive method to differentiate aggressive property of two breast cancer cell lines. Real-time impedance analysis of MCF-7 (less aggressive) and MDA-MB-231 cells (more aggressive) demonstrated unique growth pattern. Detailed slope-analysis of impedance curves at different growth phases showed that MDA-MB-231 had higher proliferation rate and intrinsic resistance to cell death, when allowed to grow in nutrient and space limiting conditions. This intrinsic nature of death resistance of MDA-MB-231 was due to modulation and elongation of filopodia, which was also observed during scanning electron microscopy. Results were also similar when validated by cell cycle analysis. Additionally, wavelet based analysis was used to demonstrate that MCF-7 had lesser micromotion based cellular activity, when compared with MDA-MB-231. Combined together, we hypothesize that analysis of growth rate, death resistance and cellular energy, through bioimpedance based analysis can be used to determine and compare aggressiveness of multiple cancer cell lines. This further opens avenues for extrapolation of present work to human tumor tissue samples.

## Introduction

Despite current advancement in technology and increasing experimental evidences, scientists are still finding it hard to tackle cancer and discover appropriate treatment regimen. One of the major concerns behind the problem is the heterogeneous nature of cancer and multiple signaling involved in regulation of cancer cells^1,2^. Heterogeneity has also been the major reason for treatment inefficacy and its failure. Widely known characteristics associated with cancer defined as hallmarks of cancer includes capability of sustaining proliferative signals, immortality, resistance to cell death, evasion to growth suppressing signals, angiogenesis and metastasis^3^. Since cancer is a heterogeneous population comprising of cells having different level of propensity towards various hallmarks of cancer, the cell type can be divided onto aggressive and non-aggressive categories. Aggressive tumors are associated with poor prognosis, therapeutic resistance and lesser human survival rate^4^. On cellular level aggressive cells can be thought of having over-expression of most of the characteristics hallmarks than lesser aggressive cancer cells. One of the important characteristics of over-aggressive cells is their ability to metastasize more and grow at a faster rate with lesser response to chemotherapeutic drugs. Although the term “aggressive” is not a specific term used to describe specific condition in cancer, but is a more general term often used by doctors and researchers to differentiate it from lesser aggressive form of cancer, as has also been defined by national cancer institute. Currently, scientists are trying to find a method of detection of cancer based on its aggressiveness^5^. Attempts are also being made to characterize aggressive cancers^6^ in molecular and genetic^7,8^ levels for combating chemoresistance^9^. ATCC (American Type Culture Collection) maintains hundred or more breast cancer cell lines and more than fifty are well characterized. Because of the heterogeneous nature of the breast cancer^10^, these cells differ with respect to their growth pattern, responsiveness to hormone, response to drugs, origin of tissues and genetic mutations^11^. Due to its complex nature, group of studies are conducted from patient’s tumor tissues for complete characterization and better understanding of the disease in genetic level^12–14^.

From the literature survey, it is perceived that cells from the same type of cancer may exhibit different characteristics with respect to their response to drug, growth rate and ability to metastasize and therefore, can be compared holistically on the basis of aggressiveness. Although tumour tissue of patients is graded using histopathology, which is also a measure of aggressive tumor, there is no thumb rule or specific assay to compare the aggressiveness of different cell lines. Therefore, quantitative assessment of cancer aggressiveness is an important requirement for therapeutics. Present investigation initiated a research study to develop a method to compare the aggressiveness of the cancer cells primarily on the basis of their growth rate and resistance to cell death. Compared to conventional various standard bioassay, the present approach follows an alternative path exploiting bioimpedance characteristics of the cells to monitor and compare the aggressive nature of breast cancer cell lines in a non-invasive and cost-effective manner. Measurement of frequency dependent variation of resistance and capacitance across electrodes containing the live cells will provide the equivalent signature of the cells which is further analyzed to evaluate the physiological condition of cell. Typically, the bioimpedance of a group of growing cells has been measured using microelectrodes which was at first introduced by Giaever and Keese^15^ as electric cell-substrate impedance sensing (ECIS) technique. The electric field lines are modulated as cells attach and start to grow on the electrode surface with the progression of time, which is reflected on the measured bio-impedance data^15^. The impedance based assay has the potential to provide time-dependent dynamic information of cellular growth events such as cell proliferation, confluence and death^16–18^. However, there is limited attempt to characterize the cancer aggressiveness based on bio-impedimetric measurement. The impedance spectroscopy is mainly focused to characterize cancer and normal cells^19^ or to study the cytotoxicity effect in real-time^20–22^. It has also been found that the real-time bioimpedance data very often employs fluctuations of impedance value which are correlated with cellular micromotion (movement of lamellipodia, filopodia, podosomes) by various groups^23–26^. Earlier, people have employed different signal processing tools like First Fourier Transform (FFT)^26^, short-time Fourier Transform (STFT)^27^, etc., to analyze these impedance fluctuations. However, there are limited works to characterize the cancer cells based on their inherent properties such as micromotions, which is related to aggressive potential of cancer cells.

With the above motivation, two breast cancer cell lines (MCF-7 and MDA-MB-231) with distinctly different growth pattern as well as therapeutic resistance^28^ have been chosen in the present research study to compare their aggressiveness through bioimpedance study. It is well known that MDA-MB-231, which is a hormone independent cell line, is highly aggressive cell line with higher growth rate, metastatic ability and resistance to drugs. On the other hand, MCF-7 cells are less invading, relatively slow growing and hormone dependent cell line^29^. Efficacy of the present method to differentiate their aggressiveness primarily concerning their growth rate and intrinsic resistance to death has been established with the help of bioimpedance analysis supported with appropriate experimental evidences.

## Results and Discussion

### Real time impedance measurement of MCF-7 and MDA-MB-231

**Figure 1:**
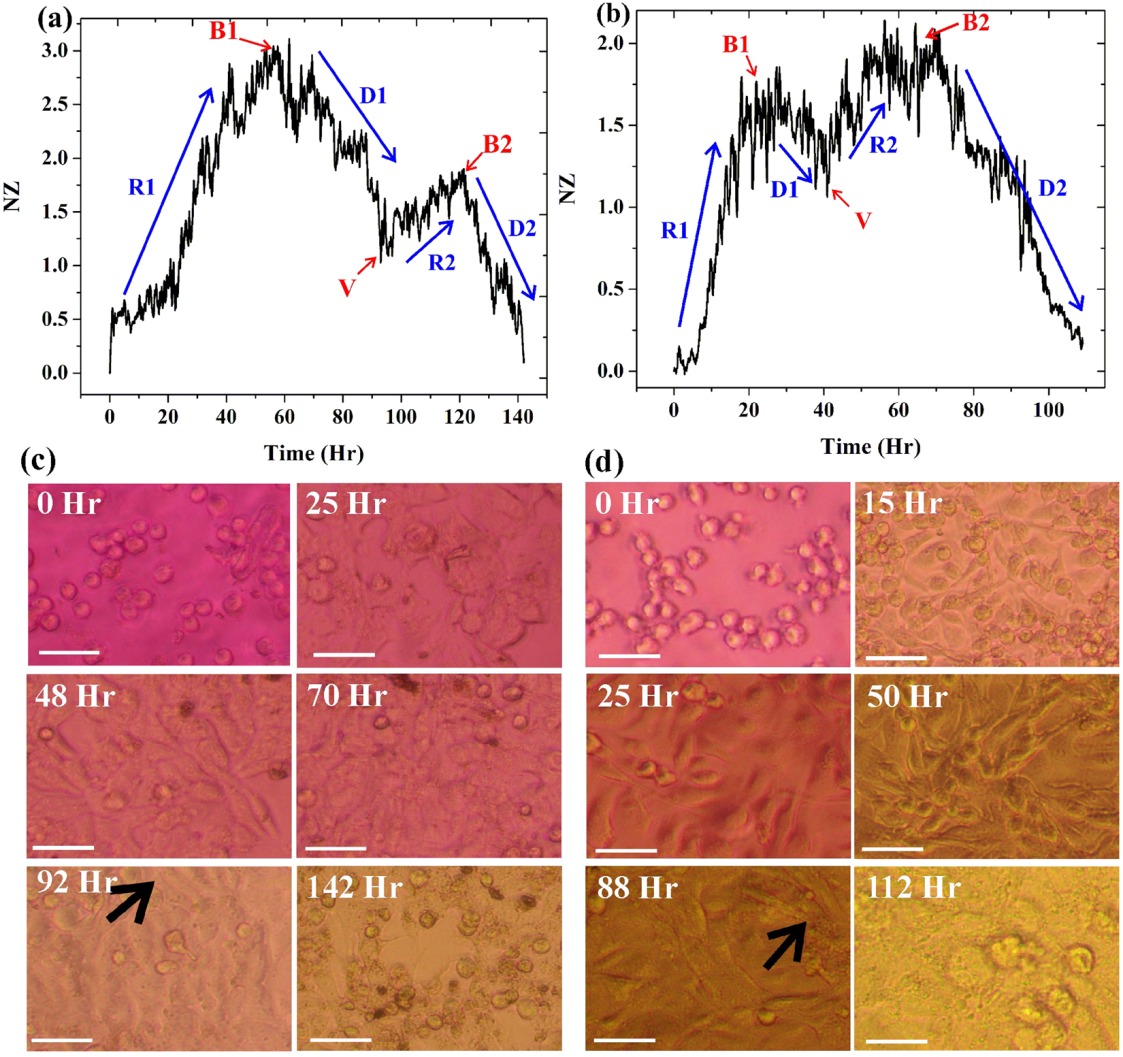
Real time impedance measurement of MCF-7 and MDA-MB-231. Real-time measurements of impedance dynamics during cell culture of (**a**) MCF-7 and (**b**) MDA-MB-231 cells. (**c**) and (**d**) are phase contrast images at different time interval for MCF-7 and MDA-MB-231, respectively. R1-rise 1, R2- rise 2, D1- death 1, D2- death 2, B1- bell 1, B2- bell 2 and V-valley point. Black arrow indicates the filopodia. Scale bar- 50 µm.

Two different human breast cancer cells MCF-7 and MDA-MB-231 cells were cultured inside the ECIS device and real-time impedance of the growing cells were measured without any medium change. Figure 1a,b show the normalized difference impedance (NZ) reflecting the growth pattern of both the cells MCF-7 and MDA-MB-231, respectively. The measured impedance was normalized at each time point with the initial impedance value just before adding the cells to the media. Figure 1c,d show the phase contrast images of both the cell lines at different time-points during the whole impedance measurement. In order to validate the impedance based cell growth assay, cell growth kinetics was also measured by manual cell counting method. The increase in impedance is mainly influenced by number of live cells attached to the growing substrate. Thus, cell number at different time interval was counted under microscope for both the cell lines and corresponding growth kinetics was plotted as shown in supplementary Fig. S1. The graph depicts all three phases of growth including a log phase, a stationary phase and a decline phase. The normalized impedance curve depict that MCF-7 cells have higher impedance value in their confluence stage as compared to MDA-MB-231 cells. From the evidence of published literature, it is understood that MCF-7 resembles luminal type cells which are well differentiated and form compact structures, whereas MDA-MB-231 looks like basal cells that form loose structure similar to mesenchymal cells^11^. Therefore, it is expected that MCF-7 cells exhibited denser tight junctions as well as higher membrane capacitance than that of MDA-MB-231 cells, hence influencing in increased NZ value for MCF-7 cells. Figure 1a,b, both the breast cancer cell lines show two successive bell shaped patterns of impedance change and do not follow the regular structure of growth curve as shown in supplementary Fig. S1. In Fig. 1a,b initial rise (R1) in impedance is due to cell adhesion, growth and division as also portrayed in phase contrast images at 25 hr and 15 hr in Fig. 1c and d for MCF-7 and MDA-MB-231, respectively. This is then followed by a phase of saturation (B1) and subsequently decrease (D1) of NZ as marked in Fig. 1a,b. Decrease in impedance is mainly due to 90–100% confluence and initial death of cells with contact inhibition as confirmed from microscopic images at 70 hr and 25 hr for MCF-7 and MDA-MB-231 cells. The graph again rises (R2) after initial death due to availability of nutrient and space and supported by inherent cellular adaptation in stress condition by forming elongated filopodia, lamellipodia. The inference drawn from the bioimpedance results is corroborated by phase contrast microscopic photographs in Fig. 1c and d taken at 92 hr and 88 hr time for MCF-7 and MDA-MB-231, respectively during cell culture experiment. The observations reveal that breast cancer cells when grown in space and nutrient limiting condition does not switch to irreversible death mode, rather they try to resist death or/and proliferate after initial death of part of the total cell population. After R2 phase, final cells death phase (D2) starts due to non-availability of nutrients and impedance value decreases to a minimum value. It is also depicted from Fig. 1a,b that MCF-7 and MDA-MB-231 cells have significantly distinct nature of impedance rise and decrease pattern, e.g. in case of MDA-MB-231 cells the first valley point (V-point) arises faster (~45 hr) than that of MCF-7 cells (~95 hr). Secondly, MDA-MB-231 cells have lower value of first bell (B1) in comparison to its second bell (B2), which is totally reverse in MCF-7 cells. Third, length and slope of D1 and D2 also varies in both cell lines as duration of D1 is ~35 hr in MCF-7 whereas, duration of D2 is ~15 hr for MDA-MB-231. Further, it can be observed that a greater fluctuation in impedance change is involved in MDA-MB-231 as compared to MCF-7. Therefore, the above observations related to impedance variation during entire cell culture process provides sufficient information about growth dynamics of cancer cells including their kinetics and which are explored further to differentiate aggressiveness of two cell lines.

### Characterization of growth dynamics to detect aggressiveness

#### Characterization of rate of cell proliferation and cell death

**Figure 2:**
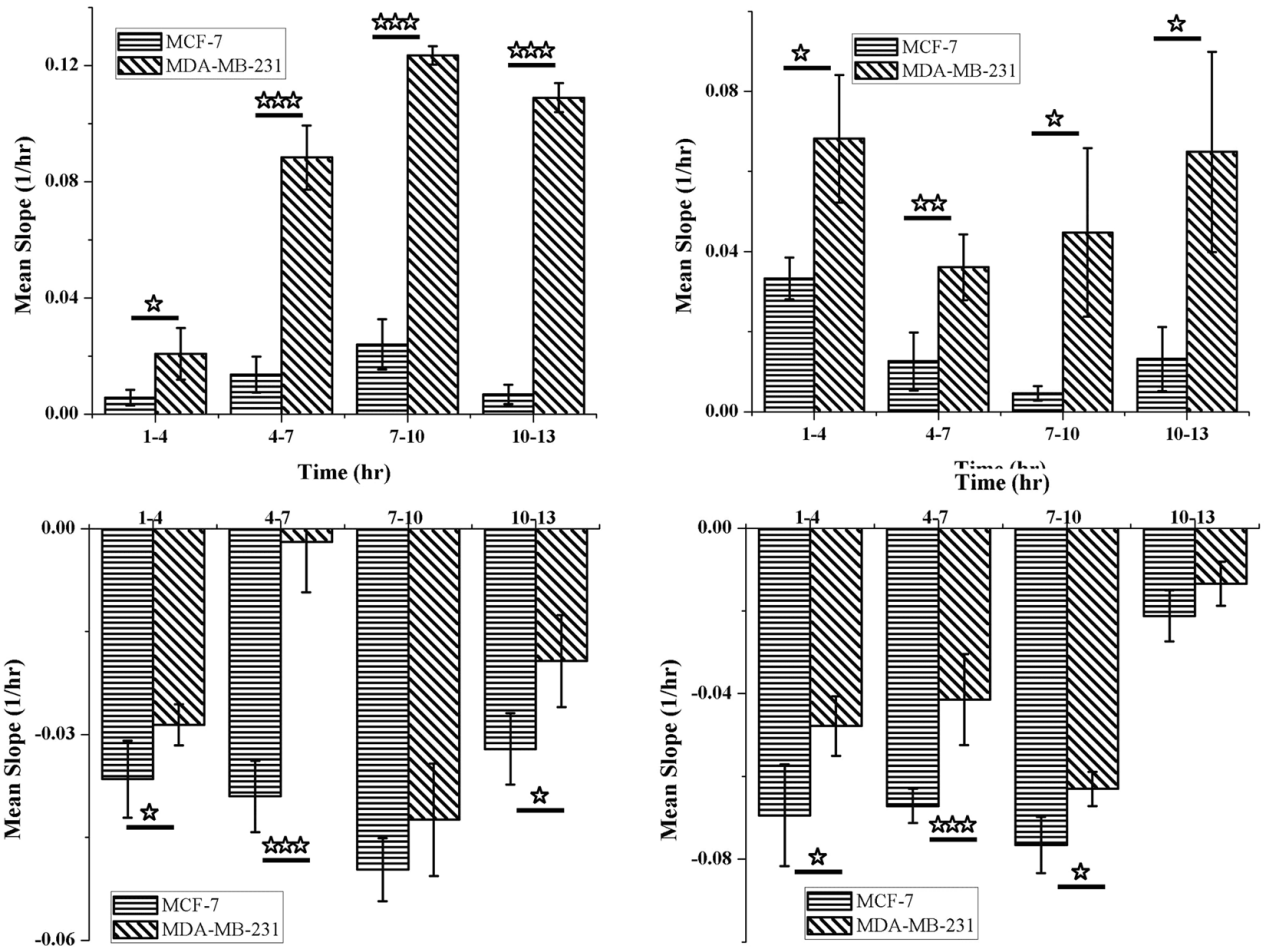
Characterization of rate of cell proliferation and cell death. The rate of proliferation and death as determined by analysing the slope of the line at 3 hr interval during phases of (**a**) Rise 1 (R1), (**b**) Rise 2 (R2), (**c**) Death 1 (D1) and (**d**) Death 2 (D2).

The impedance based results are not only comparable with the traditional growth kinetic behavior as described above but also provide more significant signature of growth kinetics. The growth dynamics as observed in Fig. 1 for different phases differ significantly for more aggressive cancer cells in comparison to less aggressive cells. Since the aggressiveness can be correlated with cell proliferation rate and as well as with resistance to cell death, slopes of the growth curves were calculated by analyzing the slope of the impedance response curve at 3 hr-interval in all the four phases (R1, D1, R2 and D2) for a total period of twelve hours. Figure 2 shows the slopes of growth curves for both MCF-7 and MDA-MB-231 cells. The result depicts that during both the rise phases (R1 and R2), the slope of the curves for MDA-MB-231 cells are always higher than that of MCF-7 cells supporting the rapid proliferation potency of MDA-MB-231 cells. Further Fig. 2c,d illustrates that during death phases (D1 and D2) the slant of the curves for MDA-MB-231 cells is constantly lower as compared to MCF-7 cells. The lower value of decline slope signifies the higher resistance to death of MDA-MB-231 (through filopodia extensions) in limited nutrient and space. Further, keeping the information at 1–4 hr’s as a control, the fold change of the other three time intervals of both the cell lines have been computed and shown in Supplementary Fig. S2. The fold change of slopes during the cell growth and cell death of MCF-7 and MDA-MB-231 cell lines again confirms the aggressiveness of MDA-MB-231 cells.

**Figure 3:**
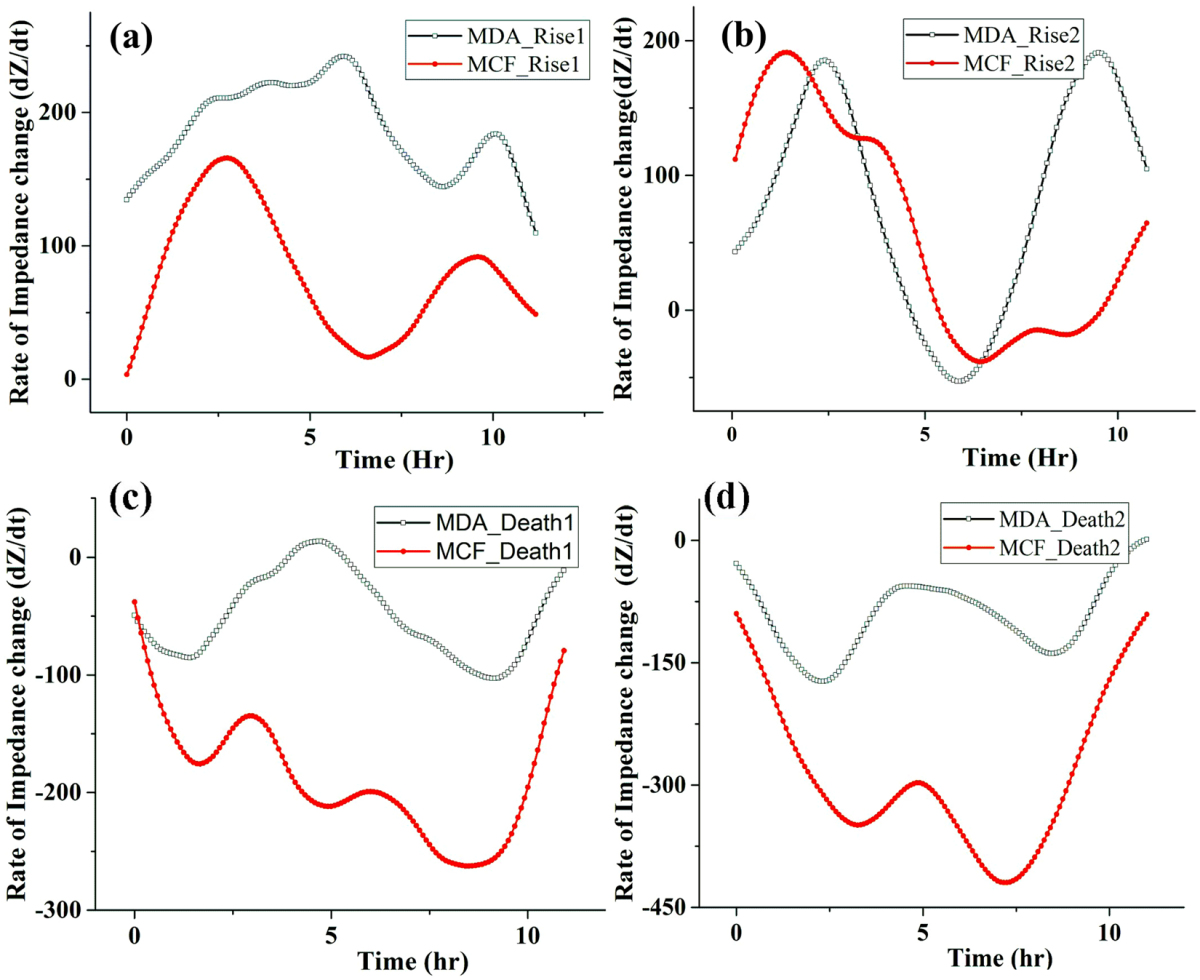
Rate of impedance during various stages of growth. Variation of rate of impedance change with time during phases of (**a**) Rise 1 (R1), (**b**) Rise 2 (R2), (**c**) Death 1 (D1) and (**d**) Death 2 (D2).

Moreover, for detail analysis of growth pattern the rate of change of impedance with time (dZ/dt versus t) was computed according to Eq. 2 for same time (12 hr) duration of different cell culture phases and results are summarized in Fig. 3. The rate of impedance change during log-phase of MDA-MB-231 cells have predominantly higher value as represented in Fig. 3a. Additionally, although the second rise is mostly dependent on availability of free space, the overall speed of impedance alteration i.e. rate of cell proliferation is higher for MDA-MB-231 cells as shown in Fig. 3b. Further, the slower decrease of impedance of MDA-MB-231 cells in death phases D1 and D2 confirms the characteristics nature of higher resistance to death process of MDA-MB-231 cells. These results confirm the ability of MDA-MB-231 cells to resist cell death more than MCF-7 under limiting conditions. The results are well concurrent with the micrographs showing cellular adaptations during death phase and cell cycle analysis.

#### Cellular micromotions to determine aggressiveness of cancer cells

**Figure 4:**
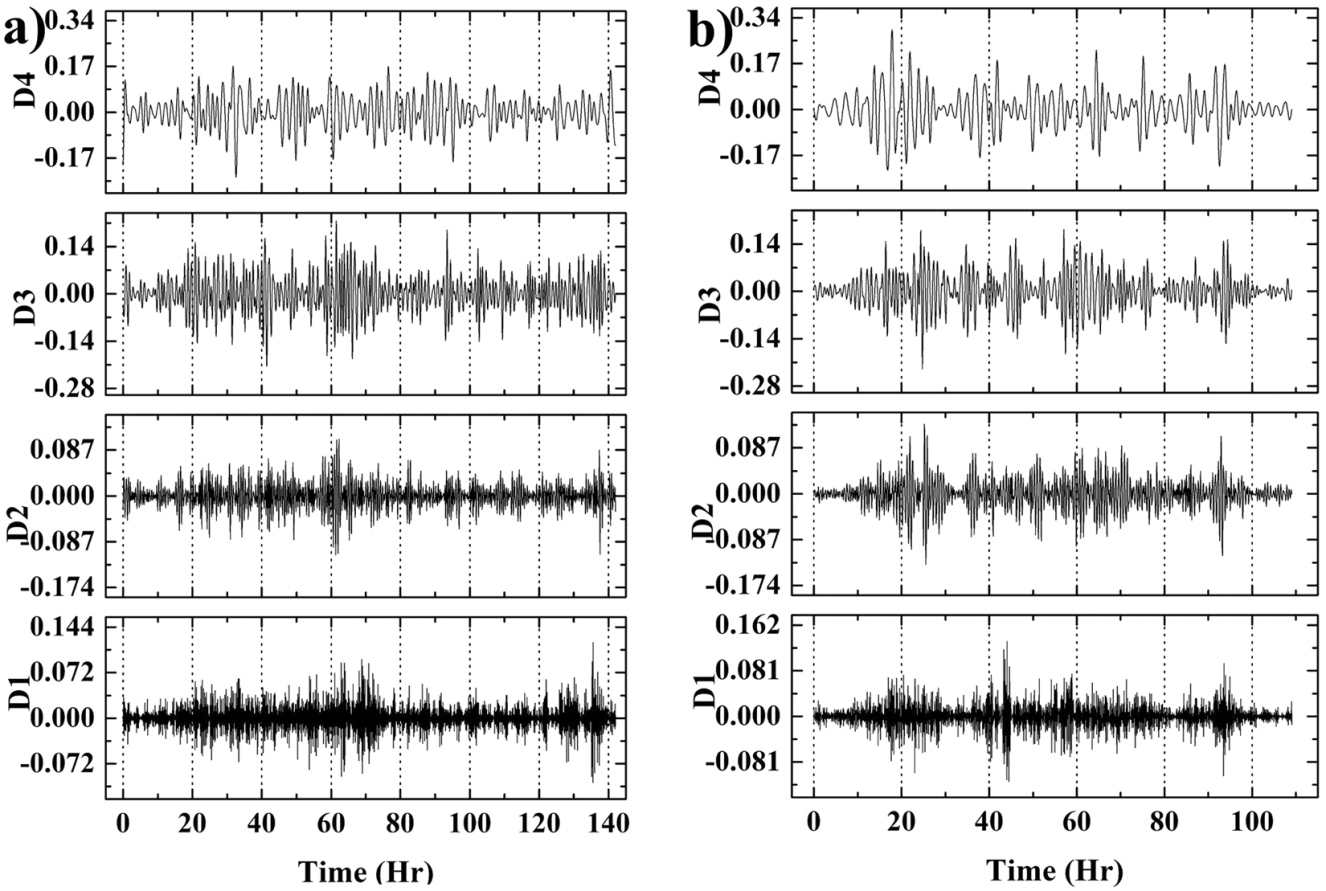
Cellular micromotions to determine aggressiveness of cancer cells. Discrete wavelet based extracted detail signal of (**a**) MCF-7 and (**b**) MDA-MB-231 cells. D1-D4 represent the decomposed detail signal at four different levels.

It has been reported in our previous study^30^ that there is a notable change in total cellular activities associated with cellular micromotions in cancer cells as compared to normal cells. The real-time measured impedance of growing cells is associated with impedance fluctuations as shown in Fig. 1. The impedance fluctuations are originated due to dynamic changes of lamellipodia, filopodia and other integrin. In our previous study, a noble wavelet based analysis technique had been established to quantify these impedance fluctuations for distinguishing cancer and normal cells^30^. The literature, it has been established that impedance spectroscopy enables to identify the behavior of adhesion kinetics and influence of associated integrin in breast cancer cells^31^ However, the study correlating the cancer aggressiveness and amount of these kinetic movement and micromotions is not explored in literature. In this section, the wavelet based study is extended to quantify the energy for cellular activities and correlate them with aggressiveness of cancer cells. The high frequency components (detail signal) were filtered out from the original signal at different levels (D1-D4) using DWT, where D4 represents highest frequency information among the four levels. Figure 4 shows the detail signal at four levels for both MCF-7 and MDA-MB-231 cells. The higher frequency components in D4 for MDA-MB-231 cells as compared to MCF-7 cells indicate existence of more long-term micromotions in MDA-MB-231 cells. These higher micromotions of cells are associated with elevated cellular activities like cell migration, division and cell-cell interactions. Further, the energy of the detail signal at different phases (R1, R2, D1, D2) for same time interval (12 hr) was calculated based on Eq. 3 to quantify and compare the cellular activities of both the cell-lines. The calculated energy values are summarized in Table 1, which indicates that MDA-MB-231 cells have significantly higher energy in all four phases. All the units in Table 1 are in arbitrary unit (a.u.) as discussed in our previous study^30^. This implies that MDA-MB-231 cells are more active throughout the time. This energy can be directly correlated with power required to execute the work done for cell-cell communications. The higher energy value even in death phase signifies that MDA-MB-231 cells are more dynamic and in the other hand possess more energy to resist the cell death. Therefore, through detailed analysis of specific phases of growth cycles helped in assigning quantitative values for the measurement of aggressiveness of two different breast cancer cell lines.

**Table 1:** Energy of the detail signal at level 3 and 4 during different phases of growth kinetics.

Phase	R1		D1		R2		D2	
	MCF-7	MDA-MB-231	MCF-7	MDA-MB-231	MCF-7	MDA-MB-231	MCF-7	MDA-MB-231
D3_E (a.u.)	0.251 ± .012	0.285 ± .014	0.267 ± .013	0.371 ± .019	0.245 ± .012	0.417 ± .018	0.224 ± .010	0.366 ± .016
D4_E (a.u.)	0.272 ± .014	0.457 ± .022	0.410 ± .020	0.514 ± .025	0.308 ± .013	0.413 ± .020	0.294 ± .014	0.346 ± .017

### Correlation of cancer aggressiveness with bioimpedance signal

#### Determination of cell viability at different growth phases

**Figure 5:**
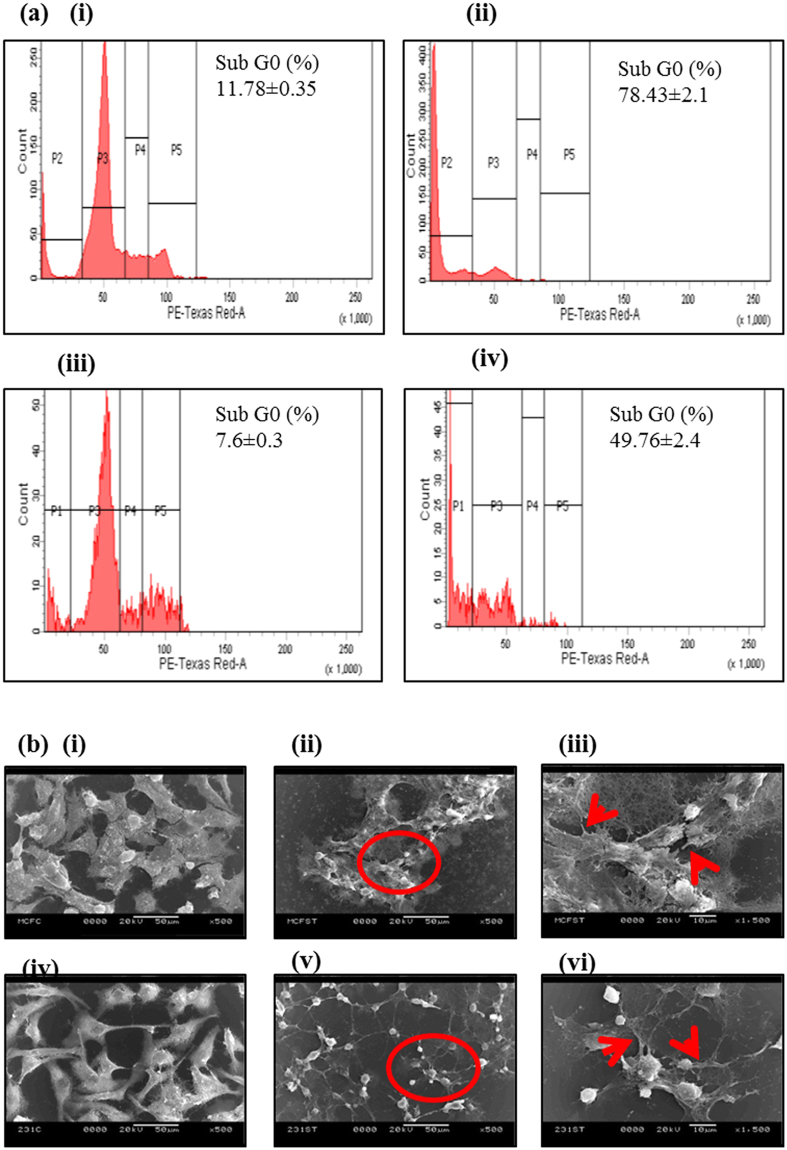
Determination of cell viability and morphology at various stages of growth phase. (**a**) Cell cycle analysis using flow cytometry of (i) MCF-7 log phase, (ii) MCF-7 death phase, (iii) MDA-MB-231 log phase, (iv) MDA-MB-231 death phase (p2 region depicts subG0 phase). (**b**) Scanning electron micrograph of (i) MCF-7 log phase, (ii) MCF-7 death phase, (iii) magnified image of circled part of (ii); (iv) MDA-MB-231 log phase, (v) MDA-MB-231 death phase, (vi) magnified image of circled part of (v). Red circle are enlarged to show magnified image. Red arrow indicates filopodia.

To determine and validate the difference between the death kinetics of MCF-7and MDA MB-231, the cell cycle analysis has been performed by commercial flow cytometry (BD-FACS Analyzer). The focus in the present experiment was to determine the percentage of cells entering in apoptotic phase (determined by sub G_0_ phase) during death phase in comparison to cells in log phase. Figure 5a (i) shows MCF-7 cells in log phase where sub G_0_ (P2) or dead cell population is about 11.78% (supplementary Table S1), whereas during death phase the sub G_0_ (P2) population rises to 78.43% as illustrated in Fig. 5a (ii). Similarly, Fig. 5a (iii) demonstrates MDA-MB-231 cells in log phase depicting sub G_0_ (P1) population to be 7.6% which rises to 49.76% in its death phase as shown in Fig. 5a (iv)-P2. Therefore, the results infer that during nutrient depletion and cells growing in a constrained area i.e. in death phase, MDA-MB-231 cells seem to resist death process more than MCF-7 correlating the decreased slope and reduced rate of impedance change as observed in Figs 2 and 3.

#### Comparison of morphology of cells in log and death phase

It has been found from bioimpedance growth study that rate of impedance change of MDA-MB-231 cells is slowed down in nutrient and space limited environment during death phase and cells adapt themselves in this stressed environment by rearranging the lamilipodial and filopodial projections as observed partially in phase contrast microphotographs shown in Fig. 1c,d. Additionally, SEM was performed to observe cytoskeleton in more detail considering cells at log phase as control. Figure 5b (i) & (iv) represent SEM micrographs of control MCF-7 and MDA-MB-231 cells, respectively demonstrating well dispersed and inter-connected cells with each other with distinct and healthy morphology. Figure 5b (ii) corresponds morphology of MCF-7 cells in start of death phase during media depletion and space constraint and cellular morphology depicts that MCF-7 cells become shrunken, smaller with dead cellular debris observed around the main cell colony. Therefore, during depletion of essential nutrient and limited space, many MCF-7 cells died and those alive are shrunk in size, indicating apoptosis as shown in Fig. 5b (iii). On contrary to stressed MCF-7 cells, MDA-MB-231 exhibited lesser cell death and demonstrated elongated and thinner filopodial structure, making contacts with adjacent cells, to form a cage like network as depicted in Fig. 5b (vi). Filopodial extensions are generally used by cells to sense external molecular cues and initiate cellular movement (i.e. migration). It is generally done with the help of bundles of actin filament directed in a single direction^32,33^. MDA-MB-231, during nutrient stress is found to increase the length of filopodia, perhaps to increase nutrient uptake and for survival. This leads to cellular migration and increased resistance to cell death. Increased filopodia is the result of elongation in the length of actin filaments. Therefore, under similar stress conditions, MDA-MB-231 cells were found to adapt and resist cell death much more than MCF-7 cells which showed more cell death in the form of dead cellular debris and shrinkage in cell size. MDA-MB-231 on the other hand adapted the stressed environment by increasing their filopodia length and getting interconnected with adjacent cells, further highlighting a way of cellular adaptation during limited nutrient and space conditions.

Therefore, the results obtained infer that real-time bioimpedance based monitoring and analysis of growth kinetics may be directly correlated with cellular growth pattern and associated change. Biologically it has been already reported MDA-MB-231 to be more metastatic and drug-resistant than MCF-7^34^. Real-time impedance measurement of both cell lines demonstrated unique growth patterns with two successive growth and death phases under limited space and nutrient conditions. The duration and nature of growth curves were unique and especially differed in proliferation and death phases and helps to differentiate their rate of proliferation and cell death without the help of any sensitive molecular assay. More aggressive cancer cell (MDA-MB-231) of the two showed rapid change of impedance indicating faster cell growth, while death phase exhibited slower cell death which was also validated by cell cycle analysis. Cellular projection like filopodia developed elongation which helped MDA-MB-231 cells to adapt in the limited nutrition and space condition, resisting overall cell death. Further wavelet based analysis also showed higher energy for MDA-MB-231 in all the growth phases which can be related to its higher proliferation, migration and dynamic structural reorientation. Therefore, the present bioimpedance analysis combined with wavelet based study can be used to differentiate the aggressive property of cancer cells depending on their growth rate and intrinsic death resistance.

## Conclusions

In the present work, bioimpedance based noninvasive technique has been employed to analyze the aggressive behavior and further differentiate the aggressiveness of cancer cells through analysis of the growth curves. The measured bioimpedance assay captures the growth kinetics in space and nutrition constrained condition and provides significantly distinct nature of impedance rise (two rise phases) and decrease (two death phases) pattern which is quite difficult to acquire using manual cell kinetic analysis. The aggressiveness of cancer cells has been correlated with cell proliferation rate, cell death resistance and cellular energy associated with micromotions. The detailed examination of impedance based growth curves in association with flow cytometry, phase contrast and SEM analysis demands the potency of bioimpedance study to characterize the aggressiveness of cancer cells. MDA-MB-231 cells have higher rate of impedance change in log phase and slower decrease in impedance change or slope in death phase as compared to MCF-7 cells. Though MDA-MB-231 cells were regarded as faster growing and having metastatic potential, it was also more resistant to apoptosis induced by depletion of nutrition and growth factors in a constrained space. The wavelet based analysis of time-series impedance data indicates that bioimpedance study with detail analysis can help to explore the micromotions associated cellular activities with detail dynamics of cells growing in 2-D *in-vitro* conditions. The higher frequency decomposed signal of both the cell lines depicted that MDA-MB-231 cells exhibited higher cellular energy correlating with its enhanced capability of cellular micromotions. Overall the growth rate, intrinsic resistance to cell death and cellular energy has been used as parameter to compare aggressiveness of both the cell lines. Therefore, the present analysis opens a new horizon for developing an automated real-time measurement system for comparing aggressiveness of different cancer cells *in-vitro* as well as from patient derived tumor samples of different grades.

## Materials and Methods

### Cell line and cell culture

MDA-MB-231 and MCF-7 cells were purchased from National Centre for Cell Science (Pune, India) and cells were cultured and maintained at 37 °C in an atmosphere with 5% CO_2_ and 95% humidity as detailed in previous literature^35^.

### Impedance measurement

An ECIS based bioimpedance sensor having eight separate culture wells was used to monitor the impedance of cells. Mini-culture well consisting of a working electrode and a common counter electrode had been fabricated in-house using microfabrication technology. Here, Agilent precision impedance analyzer 4294-A interfaced with computer was utilized for measurement of impedance change in between working and counter electrodes. The detail experimental procedures had been described in our previous study^36^. Cell concentration was diluted to 60,000 cells in 400 µl of fresh media and seeded inside the well after proper cleaning of the individual well. Subsequently, the ECIS device was kept inside the CO_2_ incubator and necessary electrical connection was been made to interface the device with the impedance analyzer. As the cells started attaching on the electrode surface and initiated to grow, the applied electric field was altered leading to change in the recorded impedance value. In the present study, the impedance of the growing cells was measured at frequency of 40 kHz with 10 mV excitation potential at 5 min time interval. All the experiments were repeated three times and average impedance values have been taken for the analysis.

### Growth kinetic measurement

Equal number of cells (190000) were seeded onto 6 well-plate maintaining similar cell density and culture media. Cells were allowed to grow under normal optimum conditions, mimicking similar conditions same as during bio-impedance measurement. After every 24 hours, media was taken out and live cells attached were detached by using 0.5% Trypsin EDTA and were manually counted by trypan blue staining under haemocytometer. A graph was plotted as normalized cell number versus time in origin.

### Monitoring cell growth phases

Cell growth was monitored in real-time by measuring the impedance of the growing cells and recorded real-time impedance data were exported to Matlab (Mathworks) for analysis. For the sake of comparison and better visibility of growth curve for both the cells, the measured impedance was normalized at each time point with the initial impedance value (*Z*_0_) as follows:

1 $$NZ=\frac{{Z}_{i}-{Z}_{0}}{{Z}_{0}}$$

where *Z*_0_ is impedance at time zero and *Z*_*i*_ is impedance at *i*^th^ time instant. The real-time normalized impedance (*NZ*) reflects the impedance variation induced by morphological changes representing the growth kinetics of cells. The normalized impedance (*NZ*) was directly correlated with number of cells attaching to the electrodes and simultaneously affected by the quality of cell interactions, adherent properties between cell-cell and cell-substrate. As real-time impedance assay describes different cell-growth phases which changes with cell types, the entire growth curve was divided into different zones for detail analysis. The growth rate of cells was calculated by determining the slope of the curve in between two time points. Additionally, the rate of change of impedance with time (d*Z*/dt) was determined to infer the rate of growth as given in following equation

2 $$\frac{dZ}{dt}=\frac{{Z}_{j+1}-{Z}_{j}}{{t}_{j+1}-{t}_{j}}$$

where *Z*_j_ is impedance at *t*_j_th time instant. This rate of impedance change was correlated with aggressive property of the cells.

### Growth dynamic analysis

The real-time impedance of the growing cells is very often associated with impedance fluctuations due to cellular micromotions. These micromotions may be correlated with the cellular activities coupled with cell-cell and cell-substrate interaction which further varies with cell types. In literature different signal processing techniques such as FFT^37^, STFT^27^, wavelet^30^ have been employed to analyze and quantify the fluctuations associated with cellular micromotions. Among these technique, wavelet based approach has been found superior with the ability to distinguish normal and cancer cells^30^. In the present study, the recorded impedance of both cancer cells were decomposed into different levels having higher and lower frequency components by Discrete Wavelet Transform (DWT) tool. DWT decomposes time series non-stationary signal at different levels into its approximate and detail components by passing through low pass filters and high pass filters, respectively. In the present study, the measured impedance data of both the cells have been decomposed into four levels with approximate signals (A1–A4) and detail signals (D1–D4). In this study, the detail signals have been correlated with cellular micromotions. Now to quantify the cellular activities, the energy (*E*3, *E*4) of the high frequency signal (D3) and (D4) at 3^rd^ and 4^th^ level, respectively was calculated based on following equation and correlated with associated cellular micromotions:

3 $${E}_{x}=\sum _{j=1}^{N}{D}_{x}^{2}(n)$$

where, *x* = 3, 4 and *N* is length of the signal D4.

### Scanning Electron Microscopy (SEM)

Equal number of both cells (MCF-7 and MDA-MB-231) were seeded in a cover slip (0.8 cm × 0.8 cm) kept in a 48 well plate, and allowed to grow in DMEM media in a atmosphere of 37 °C and 5% CO_2_. Cover slips were taken out during the middle of log phase and death phase, followed by fixation with 3.7% formaldehyde for ten minutes. As explained in earlier literature^38^ cells were subsequently washed three times with PBS buffer and were subjected to series of dehydration step. Subsequently the samples were then air dried and mounted on a stub. Subsequently, they were placed in a vacuum chamber of SEM gold coating apparatus and gold was coated at 2.5 kV, 20–25 mA for two minutes. The micrographs of the cells were then observed using a scanning electron microscope (JEOL JSM-5800, Japan) using 20 kV acceleration voltage.

### Flow cytometry

The cell cycle distribution of MDA-MB-231 and MCF-7 was determined by flow cytometry according to previously described method^39^. Equal cells were seeded in a 60 mm petri-dish maintaining similar cell density with earlier experiments and were allowed to grow without changing the medium or supplementing it. Cells were collected at log phase and death phase and analyzed using propidium iodide in a flow cytometer (BD Bioscience FACS Aria (III)).

### Phase contrast microscopy

Micrographs of cells growing inside ECIS culture well were taken at different time interval during real-time measurement of bioimpedance with the help of Olympus IX51 phase contrast microscope at 100 × magnification.

## Electronic supplementary material

Supplementary information
