# Supplementary material for: Bioimpedimetric analysis in conjunction with growth dynamics to differentiate aggressiveness of cancer cells
Source: Sci Rep. 2018 Jan 15;8:783. doi: 10.1038/s41598-017-18965-9 (PMC5768811; doi:10.1038/s41598-017-18965-9)
Supplement: Supplementary file 1 — Supplementary information [file 41598_2017_18965_MOESM1_ESM.pdf]

Bioimpedimetric analysis in conjunction with growth dynamics to differentiate aggressiveness of cancer cells

Aditya Parekh,[1,a] Debanjan Das,[1,b] Subhayan Das,[a] Santanu Dhara[a], Karabi Biswas[c], Mahitosh Mandal[a,*], Soumen Das[a,*]

[a]School of Medical Science and Technology, IIT Kharagpur, West Bengal, India

[b]Department of Electronics and Communications Engineering, DSPM IIIT, Naya Raipur, India

[c]Department of Electrical Engineering, IIT Kharagpur, West Bengal, India

[1]Joint authors having equal contribution

[*]Corresponding authors

Dr. Soumen Das- sou@smst.iitkgp.ernet.in; Dr. Mahitosh Mandal-

mahitosh@smst.iitkgp.ernet.in

Supplementary Figures-

Sup-1)

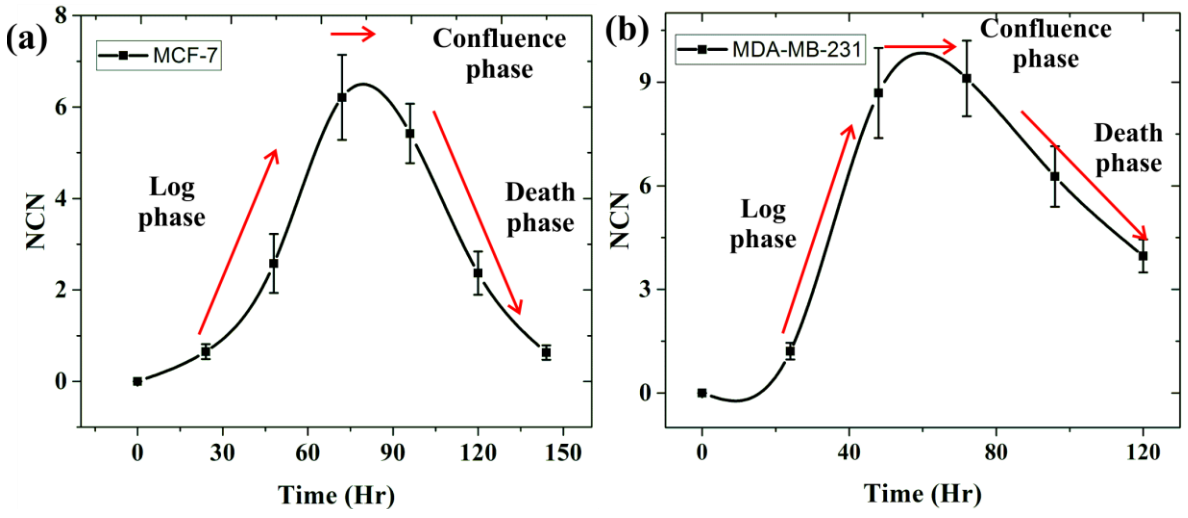

Figure S1. Growth kinetics of (a) MCF-7 and (b) MDA-MB-231. Graph depicts normalized cell number versus time representing log, confluence and death phase of cells during cells culture. The cell number is normalized with respect to initial number of cells and termed as NCN.

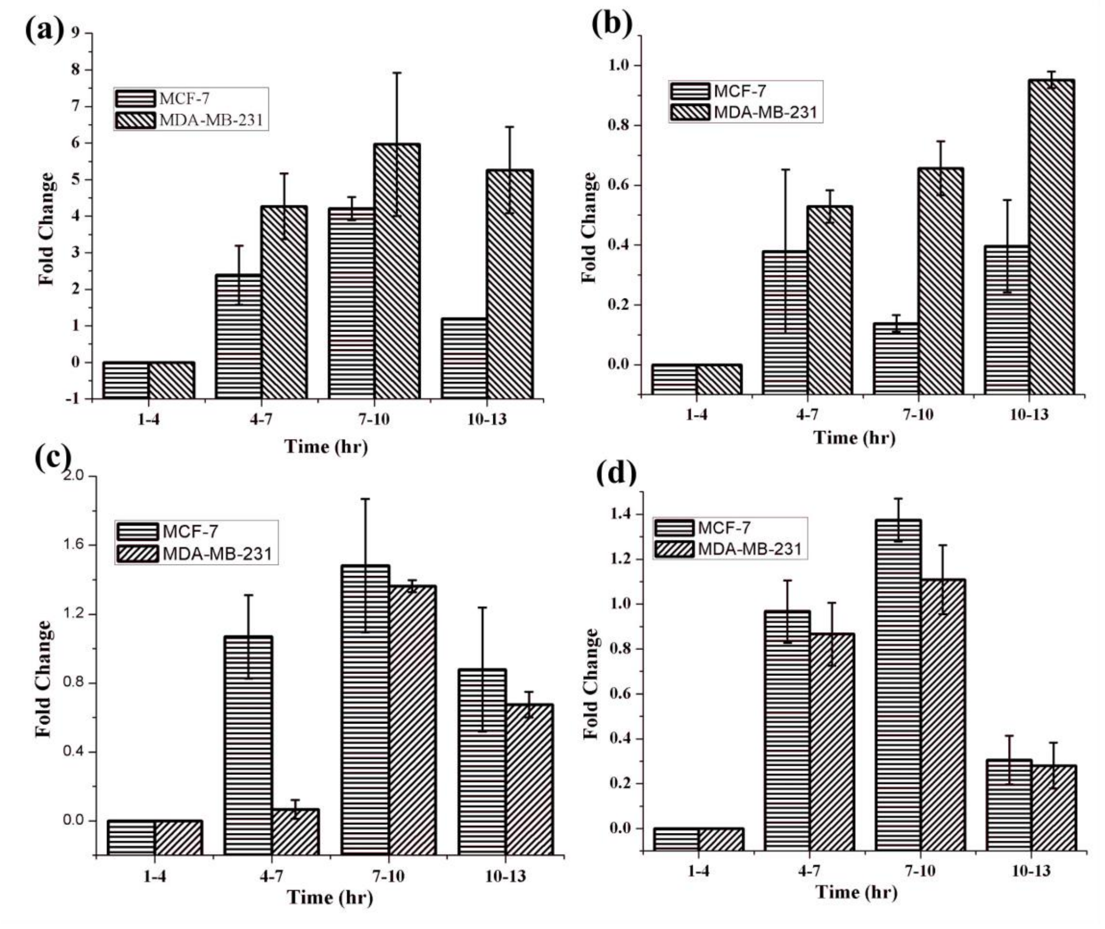

Figure S2- Fold change analysis of rate of proliferation or death both the cell line, keeping 1-4 hour as control (a) Rise 1 (R1), (b) Rise 2 (R2), (c) Death 1 (D1) and (d) Death 2 (D2).

Supplementary Table

Table-S1: Sub G_0 or dead cell population during log phase and death phase of two breast cancer cell lines (MCF-7 & MDA-MB-231) as measured in flow cytometry.

**Cell types**	**Sub G_0 population (percentage)**	
	**Log phase**	**Death phase**
MCF-7	11.78±0.35	78.43±2.1
MDA-MB-231	7.6±0.3	49.76±2.4
